# Ruthenium and Nickel Molybdate-Decorated 2D Porous Graphitic Carbon Nitrides for Highly Sensitive Cardiac Troponin Biosensor

**DOI:** 10.3390/bios12100783

**Published:** 2022-09-22

**Authors:** Walaa Khushaim, Veerappan Mani, Karthik Peramaiya, Kuo-Wei Huang, Khaled Nabil Salama

**Affiliations:** 1 Sensors Lab, Advanced Membranes and Porous Materials Center, Computer, Electrical and Mathematical Science and Engineering Division, King Abdullah University of Science and Technology (KAUST), Thuwal 23955-6900, Saudi Arabia; 2 KAUST Catalysis Center and Physical Science and Engineering Division, King Abdullah University of Science and Technology (KAUST), Thuwal 23955-6900, Saudi Arabia

**Keywords:** two-dimensional materials, nickel molybdate, ruthenium nanoparticles, cardiac troponin, aptasensor

## Abstract

Two-dimensional (2D) layered materials functionalized with monometallic or bimetallic dopants are excellent materials to fabricate clinically useful biosensors. Herein, we report the synthesis of ruthenium nanoparticles (RuNPs) and nickel molybdate nanorods (NiMoO_4_ NRs) functionalized porous graphitic carbon nitrides (PCN) for the fabrication of sensitive and selective biosensors for cardiac troponin I (cTn-I). A wet chemical synthesis route was designed to synthesize PCN-RuNPs and PCN-NiMoO_4_ NRs. Morphological, elemental, spectroscopic, and electrochemical investigations confirmed the successful formation of these materials. PCN-RuNPs and PCN-NiMoO_4_ NRs interfaces showed significantly enhanced electrochemically active surface areas, abundant sites for immobilizing bioreceptors, porosity, and excellent aptamer capturing capacity. Both PCN-RuNPs and PCN-NiMoO_4_ NRs materials were used to develop cTn-I sensitive biosensors, which showed a working range of 0.1–10,000 ng/mL and LODs of 70.0 pg/mL and 50.0 pg/mL, respectively. In addition, the biosensors were highly selective and practically applicable. The functionalized 2D PCN materials are thus potential candidates to develop biosensors for detecting acute myocardial infractions.

## 1. Introduction

Cardiovascular disease (CVD) is the most prevalent chronic disease worldwide, accounting for 17.3 million deaths per year across the globe, projected to grow to over 23.6 million deaths per year by 2030 [1,2]. CVD is a major public health concern and imposes a huge economic burden on people and governments. Cardiac troponin (cTn-I) is the most promising biomarker for CVD, and cTn-I biosensors are employed for the clinical diagnosis of CVD [3]. As the ranges of cTn-I levels in serum are in the pg/mL level, functional nanomaterials, which enhance the sensitivity and detection ranges of cTn-I biosensors, are needed to increase their performance [4,5].

Two-dimensional layered materials are outstanding materials for biosensing applications, mainly because of their large surface areas, conductivity, catalytic sites, solution processability, and ease of synthesis and functionalization [6,7,8,9]. Graphitic carbon nitride nanosheets (GCN) are emerging 2D materials known for their distinctive properties, such as defect sites, edge densities, nitrogen lone pairs, and pi-densities [10,11,12]. Porous graphitic carbon nitride (PCN) is the derivative of GCN installed with significant porous structures and altered electronic structures [13]. The surface area, structural N defect densities, and the number of surface-active sites—all crucial characteristics of a material to be exploited in biosensing applications—are increased by the existence of a porous structure [14,15]. Most importantly, the porous structures provide a high level of flexibility to the material, which enable an increased adaptability and stability of the bio-receptors that are attached to them [16,17]. Recent research showed that PCN outperforms GCN and provides a 2.8-fold increased hydrogen evolution efficiency because of the added porosity of the 2D layers [18]. PCN conductivity and aptamer immobilizing characteristics can be further improved by decorating the PCN surface with metal nanoparticles [19,20].

Ru nanoparticles (RuNPs) are interesting materials known for their excellent electrocatalytic properties [21]. Recent studies found that RuNPs exhibit a peroxidase-like behavior and they could be used as potential materials to fabricate biosensors [22]. Ru-based binary or multi-metallic NPs are employed in electrochemical sensors because of their distinctive electrocatalytic properties, better surface area-to-volume ratios, and synergistic effects [23]. Combining a porous support with RuNPs not only stabilizes the NPs but also enables synergistic properties; PCN-RuNPs have never been used in biosensing applications. Nickel molybdate (NiMoO_4_) is a binary metal oxide which provides improved redox properties of Ni and Mo for electrochemical applications [24]. Mo sites have a unique chemical affinity for thiolated aptamer linkers, bound via van der Waals force. Furthermore, the Ni and Mo atoms are well-known for their multiple redox characteristics which are useful to mediate electron transfer reactions between aptamers and electrode surfaces.

**Figure 1:**
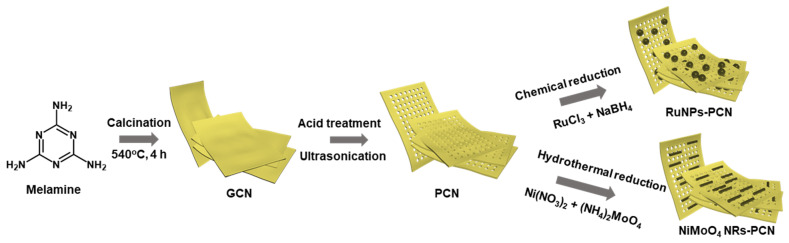
Synthesis of PCN-RuNPs and PCN-NiMoO_4_ NRs.

The objective of this work was to develop a sensitive cTn-I sensing system, using RuNPs and NiMoO_4_ nanoparticle-functionalized PCN materials. First, PCN was prepared from GCN through a simple acid-treatment. Next, RuNPs and NiMoO_4_ NRs-functionalized PCN materials were synthesized by subsequent chemical/hydrothermal treatments (Figure 1). Following that, these materials were employed to modify the working electrodes of laser-scribed graphene electrodes, which were later immobilized with cTn-I-specific thiol-functionalized aptamers to construct a cTn-I biosensor. Our investigations found that PCN-based 2D materials are excellent candidates to develop biosensors for cardiac biomarkers. The advantages of the described synthetic method are the simple and straightforward synthetic procedures, the use of melamine as a low-cost precursor, and the use of inexpensive synthesis instruments.

## 2. Experimental

### 2.1. Chemicals and Apparatus

A thiolated aptamer consisting of the DNA sequence CGTGCAGTACGCCAACCTTTCTCATGCG CTGCCCCTCTTA, developed previously by Jo et al., was purchased from Sigma and used in this work [25]. 100 µM aptamer stock solution was prepared and stored at –20 °C. A stock solution of 200 µM mercaptohexanol (MCH) was prepared in 0.01 M PBS. Myoglobin (Myo), cardiac troponin I (cTn-I), cardiac troponin T (cTn-T), and cardiac troponin C (cTn-C) were bought from Abcam (Cambridge, UK). H4522 human serum was purchased from Sigma. Scanning electron microscopy (SEM) studies were performed using Zeiss Merlin and TeneoVS field-emission scanning electron microscopes. Transmission electron microscopy (TEM) experiments were conducted using the FEI Themis Z instrument (Thermo Fisher Scientific, Massachusetts, United States). XRD patterns were performed using Bruker X-ray diffraction (XRD) D8 advance equipment (Karlsruhe, Germany). AMICUS X-ray photoelectron spectroscopy (XPS) apparatus from Kratos Analytical (Manchester, UK) was used for the XPS analysis. A multiEmStat3-PalmSens electrochemical workstation was used for the electrochemical studies. Polyimide (PI) substrates were bought from Utech Products (New York, NY, USA). For laser patterning, a CO_2_ laser from Universal Laser Systems^®^ PLS6.75 with a 150 m laser spot diameter and a 10.6 m wavelength was used [26,27]. A three-electrode system with diameters of 2.8 cm, 1.2 cm, and 1.5 mm was patterned onto a pre-cleaned PI sheet. To prepare laser-scribed graphene electrodes (LSGEs) with a relatively low sheet resistance (58/square), the following ideal parameters were used: 3.2 W power, 2.8 cm/s speed, 1000 pulses per inch, and 2.5 mm *Z* distance [28]. The electrochemical cell was a three-electrode chip system, with the reference and counter electrodes being laser-scribed graphene electrodes and the working electrode being either RuNPs-PCN or NiMoO_4_ NRs-PCN. An electrode connector was used to link the chips to the PalmSens electrochemical workstation. The cell volume was 75 µL.

### 2.2. Nanomaterials Synthesis

Figure 1 shows the synthesis of GCN, PCN, RuNPs-PCN, and NiMoO_4_ NRs-PCN. First, using a standard thermal polymerization technique, GCN was made from melamine. A GCN powder was produced by calcining 2 g of melamine at 540 °C for 4 h at a rate of 5 °C/min. The color of the powder changed from white to yellow, signifying the formation of GCN. The GCN powder was washed three times in water and three times in ethanol, and then dried at 50 °C overnight. Next, an acid treatment was implemented to prepare PCN from GCN. About 250 mg of GCN powder was added to 30 mL H_2_SO_4_, and subsequent ultrasonic agitation was performed for 15 min. The mixture was then continually agitated with a magnetic stirrer until the color of the solution changed from yellow to colorless. Finally, 100 mL of ice-cold water was added to the reaction media, producing white-colored flakes. The resulting flakes were centrifuged, washed three times with water and three times with ethanol, and then dried at 50 °C overnight.

A typical chemical reduction method was adopted to deposit RuNPs onto the PCN surface. About 0.50 g of PCN was dispersed in 50 mL water. RuCl_3_ was then added and agitated for 15 min. The mixture was then agitated for an additional 1 h after 10 mL of 0.1 M NaBH_4_ solution was added. RuNPs-PCN nanomaterial was produced by centrifuging the final product, washing it three times with water and three times with ethanol, and then freeze-drying it for an entire night. Next, NiMoO_4_ NRs-PCN nanomaterial was prepared using a hydrothermal procedure. About 0.50 g of PCN was dispersed in 50 mL of water. Then 2 mM nickel nitrate and ammonium molybdate were added to the PCN solution and the whole mixture (70 mL) was transferred to a Teflon-lined autoclave and treated hydrothermally at 170 °C for 4 h. The product was separated, washed with water and ethanol, and freeze-dried overnight, yielding NiMoO_4_ NRs-PCN.

### 2.3. Biosensor Fabrication Steps and Analysis Procedure

**Figure 2:**
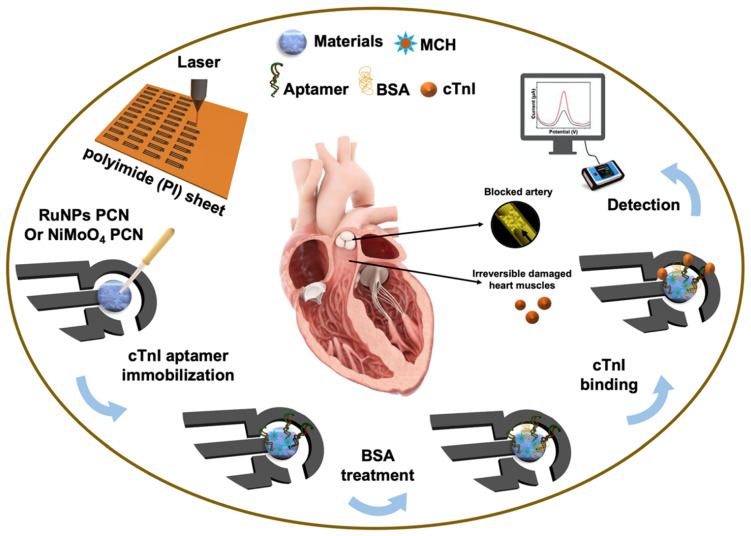
Schematic illustration for the fabrication of the RuNPs-PCN/LSGE aptasensor or the NiMoO_4_ NRs-PCN/LSGE aptasensor.

About 2 mg/mL of RuNPs-PCN or NiMoO_4_ NRs-PCN aqueous solutions were prepared by ultrasonically treating the respective dispersion for 15 min. About 5.0 µL of RuNPs-PCN or NiMoO_4_ NRs-PCN was dropped onto the working electrode of the LSGE and dried. The resulting electrodes were either RuNPs-PCN/LSGE or NiMoO_4_ NRs-PCN/LSGE. Next, the nanomaterial-modified electrodes were used to fabricate the cTn-I aptasensor, which is schematically presented in Figure 2. First, a mixture of 8.0 µM (optimized) cTn-I aptamer and 20 µM MCH was prepared in 10 mM PBS (pH 7.4). About 6.0 µL aptamer-MCH mixture was drop-casted onto the working electrode of the modified LSGEs and incubated for 12 h. The modified electrode was then washed with PBS three times. To prevent non-specific sites, the electrode was then incubated with 0.10 mg/mL BSA for 20 min. The resulting devices were either an RuNPs-PCN/LSGE aptasensor or an NiMoO_4_ NRs-PCN/LSGE aptasensor. First, desired quantities of cTn-I were produced in 10 mM PBS for cTn-I analysis. Then, 10 µL of cTn-I was placed on the working electrodes and incubated for 30 min. The electrodes were then cleaned three times with 0.01 M PBS (pH 7.40) to get rid of the unbound analytes. For real sample analysis, cTn-I-spiked human serum samples were used. 10 µL of serum sample was dropped onto the working electrode, incubated for 30 min, washed with PBS, and then analyzed using either the RuNPs-PCN/LSGE aptasensor or the NiMoO_4_ NRs-PCN/LSGE aptasensor.

### 2.4. Electrochemical Assay Conditions

Cyclic voltammetry (CV) experiments were conducted in a potential range of −0.40 V to + 0.40 V and a scan rate of 0.05 V/s, unless otherwise specified separately in the figure captions. Squarewave voltammetry (SWV) studies were carried out with a 2 Hz frequency and 0.10 V amplitude. Electrochemical impedance spectroscopy (EIS) analysis was used with a frequency range of 1.0 Hz–100 kHz, an amplitude of 0 V, and a bias potential of 5.0 mV. The calibration plots were prepared using normalized current, which was determined by subtracting the peak currents of the redox probe before and after cTn-I binding. All the electrochemical experiments were conducted in 0.10 M KCl containing a mixture of 5.0 mM K_3_/K_4_[Fe(CN)_6_]. All measurements were repeated at least three times and the average was used to prepare data.

## 3. Results and Discussions

### 3.1. Characterizations

**Figure 3:**
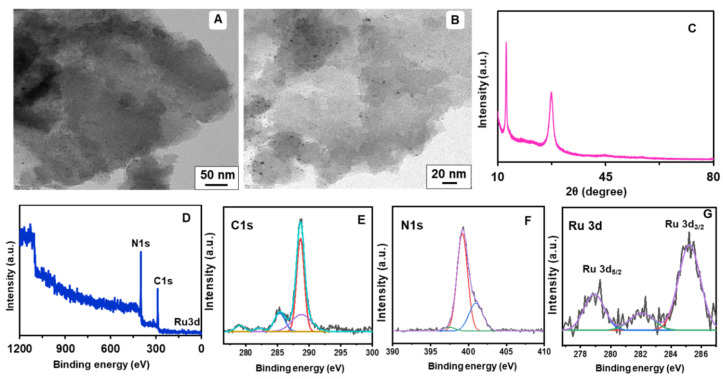
TEM images (**A**,**B**), XRD pattern (**C**), XPS survey spectrum (**D**), and XPS deconvoluted spectra (**E**–**G**) of PCN-RuNPs.

**Figure 4:**
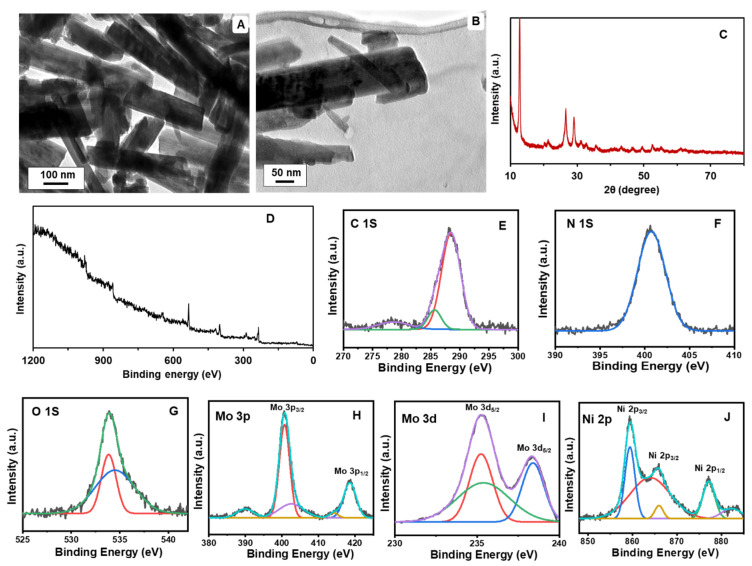
TEM images (**A**,**B**), XRD pattern (**C**), XPS survey spectrum (**D**), and deconvoluted XPS spectra (**E**–**J**) of PCN-NiMoO_4_ NPs.

The TEM analysis of the PCN-RuNPs showed a large number of porous structures with a distribution of ultrafine Ru nanoparticles (Figure 3A,B). The particle size of Ru was in the range of 1–3 nm. The XRD pattern of PCN-RuNPs showed the typical crystal characteristic of carbon nitride (Figure 3C). PCN crystal structure was confirmed by two prominent peaks observed at 3.3° and 27.0°, which were associated with (100) and (002) planes, respectively [29]. The XRD curve of PCN-RuNPs did not show any typical peaks of the Ru phase, which is likely due to the ultra-small particle size and high dispersion degree of RuNPs in the material. XPS was then used to analyze the surface electrical structure of the PCN-RuNPs (Figure 3D–G). The XPS survey spectrum showed signals that matched the elements C, N, and Ru. For the C 1s spectrum, moderately overlapped with the Ru 3d region, the divided peaks at 285.8, 284.8, 282.9, and 280.7 eV were assigned to N–C=N, C–C, Ru° 3d_3/2_, and Ru° 3d5/2, respectively. Two usual bands for Ru 3d_3/2_ and Ru 3d_5/2_ were also observed, consistent with previous reports [30]. The TEM image of PCN-NiMoO_4_ NPs revealed the distribution of nanorod-shaped NiMoO_4_ particles (Figure 4A). The nanorods were interconnected within the backbone of the PCN sheets (Figure 4B). The sheet-like structure of PCN was not evident in the TEM image because the NiMoO_4_ structure darkened the PCN sheets. The crystal characteristic of the PCN phase and NiMoO_4_ were perceived in the XRD pattern of PCN-NiMoO_4_ NPs (Figure 4C). The XRD pattern is indexed in accordance with JCPDS file # 31-0902, which corresponds to the monoclinic phase of α-NiMoO_4_ [31]. In addition, characteristic PCN bands of 13.4° and 27.8° were identified. The XPS survey spectrum displayed the expected elements of C, N, O, Ni, and Mo in the composite (Figure 4D–J). The spectra of C 1S, N 1S, and O 1S were consistent with the corresponding pattern of PCN. The core-level spectrum of Ni 2p showed two key peaks at 856.5 and 878.7 eV, belonging to Ni 2p_3/2_ and Ni 2p_1/2_, respectively, which are features of the Ni^2+^ oxidation state. The deconvoluted Mo 3d core-level spectra had doublet peaks at 232.5 and 235.8 eV which corresponded to Mo 3d_5/2_ and Mo 3d_3/2_, respectively, and showed that Mo was in the Mo^6+^ state. In the O 1s binding energy spectrum, the peak at 530.5 eV represented metal–oxygen bonds. The Mo/Ni atomic ratio was found to be around 1:1.5, implying that some amorphous Ni may have co-existed in the structure [32].

### 3.2. Electrochemically Active Surface Area

**Figure 5:**
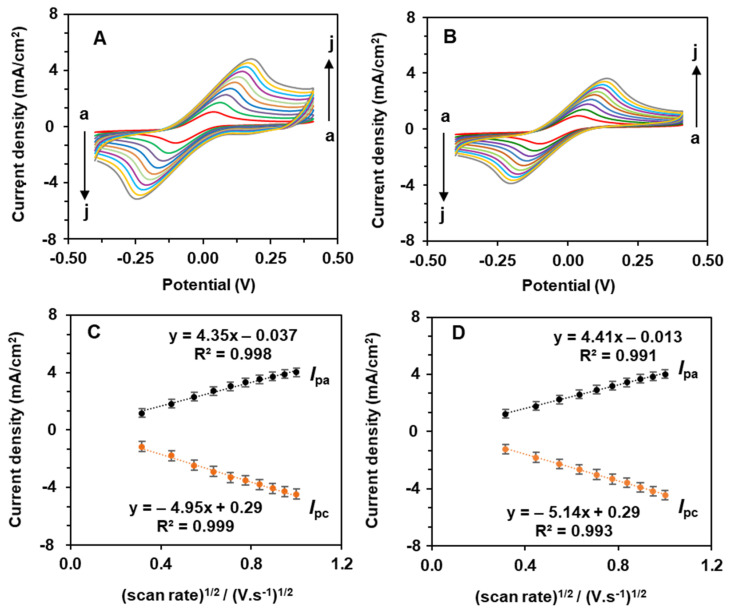
CVs for PCN-RuNPs (**A**) and PCN-NiMoO_4_ NRs (**B**) modified LSGEs were obtained using 0.10 M KCl containing 5 mM K_3_/K_4_[Fe (CN)_6_] at various scan rates from 0.10 to 1.0 V/s (a to j). Peak currents for PCN-RuNPs (**C**) and PCN-NiMoO_4_ NRs plotted against square root of scan rate (**D**).

The electrochemical properties of the 2D nanomaterials were tested with the ferri/ferrocyanide redox model system (Figure 5A–C). The CVs of the PCN-RuNPs and PCN-NiMoO_4_ NRs showed the typical redox couple of Fe^2+^/Fe^3+^. However, the kinetics of the redox process varied which is evident from the different slope values obtained in their kinetic plots (Figure 5D–F). Furthermore, the electrochemically active surface area (EASA) values of the electrodes were assessed by substituting the slope value of the kinetic plot into the repositioned Randles-Sevick equation Equation (1),

(1) =IPaν[1268600 n32 D12 C]

Here, *A* = electrochemical active surface area of the electrode, *D* = diffusion coefficient (6.70 × 10^−6^ cm^2^.s^−1^), *v* = scan rate (V.s^−1^), *C* = concentration of ferri/ferrocyanide solution (mol·cm^−3^), and *I*_p_ = peak currents (µA). The EASA values of PCN were also calculated for comparison.

The EASAs of the PCN-RuNPs, PCN-NiMoO_4_ NRs, and PCN were estimated to be 0.0894, 0.0917, and 0.0872 cm^2^, respectively. The EASA trend follows the pattern of PCN-NiMoO_4_ NPs > PCN-RuNPs > PCN. The values of the EASAs were significantly increased compared to their geometrical area. Both PCN-RuNPs and PCN-NiMoO_4_ NRs showed an increase in performance compared to PCN. The considerable increase in the values of the EASA was because of the electrocatalytic properties of the RuNPs, NiMoO_4_, and PCN materials. The major contact between PCN materials and the LSGE surface was most likely the result of a pi-stacking interaction between the triazine ring densities in PCN and the delocalized electrons in graphene sheets [33].

### 3.3. Aptasensing Performances

**Figure 6:**
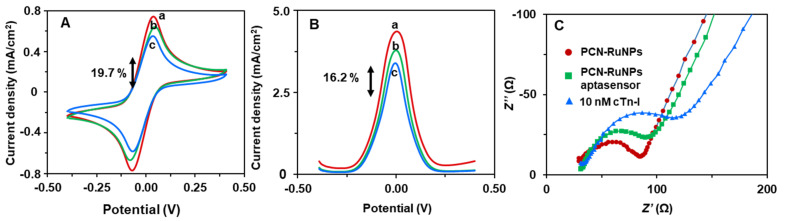
(**A**) CV, (**B**) SWV, and (**C**) EIS curves of PCN-RuNPs (a), PCN-RuNPs aptasensor (b), and PCN-RuNPs aptasensor + 10 ng/mL cTn-I (c). The supporting electrolyte 0.10 M KCl containing a mixture of 5.0 mM K_3_/K_4_[Fe(CN)_6_].

Next, the biosensing aptitudes of the PCN-RuNPs and PCN-NiMoO_4_ biosensors were tested. Figure 6 presents the CV, SWV, and EIS responses of the PCN-RuNPs aptasensors with and without cTn-I. The responses of the PCN-RuNPs material were also included for comparison. 10 ng/mL cTn-I elicited a significant decrease in the redox signals of the probe, indicating that the biosensor has the ability to sense cTn-I. The decrease was about 19.7% in CV and 16.2% in SWV. The decrease was because of the specific binding of the cTn-I with the recognition regions of its aptamer. This specific analyte-aptamer binding event inhibits the flow of electrons between Fe^2+^/Fe^3+^ ions and the PCN-RuNPs/LSGE, which was revealed as a decreased signal in the current response. Evidently, the flow of electrons would be slowed down more rigorously if high concentrations of cTn-I are present, thus allowing a relationship between the concentration of the cTn-I and the normalized current signal of the redox probe. In addition to CV and SWV, EIS was used to further examine the sensor because EIS is a highly sensitive tool to look at changes in the interfacial electron transport. The charge transfer resistance of the PCN-RuNPs sensor was significantly increased in the presence of cTn-I. Apparently, the aptamer-cTn-I binding event increased the resistance for the interfacial electron transport caused between the aptamers and cTn-I, which resulted in the increased charge transfer resistance (*R*_ct_).

**Figure 7:**
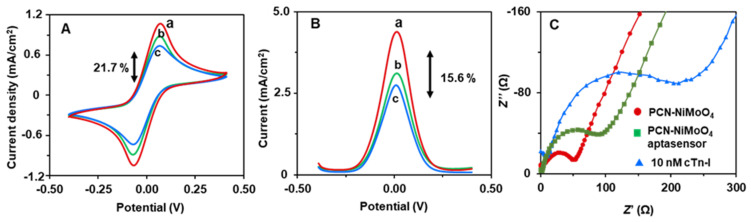
(**A**) CV, (**B**) SWV, and (**C**) EIS curves of PCN-NiMoO_4_ NPs (a), PCN-NiMoO_4_ NPs aptasensor (b), and PCN-NiMoO_4_ NPs aptasensor + 10 ng/mL cTn-I (c). The supporting electrolyte 0.10 M KCl containing a mixture of 5.0 mM K_3_/K_4_[Fe(CN)_6_].

The sensing results of the PCN-NiMoO_4_ aptasensor are similar to the PCN-RuNPs aptasensor, but the sensitivity is relatively higher for the PCN-NiMoO_4_ NRs (Figure 7). The decrease is about 21.7% in CV and 15.6% in SWV. Because the NiMoO_4_ has a relatively excessive EASA, edge densities, and redox properties originated from the advantage of having a binary composition of metal oxides, i.e., nickel oxide and molybdenum oxide. Overall, both the PCN-RuNPs aptasensor and the PCN-NiMoO_4_ NRs aptasensor showed a significantly improved biosensing performance for the quantitative detection of cTn-I. A variety of interactions operate between PCN materials, aptamers, and cTn-I molecules. A biomimetic microenvironment for the incoming aptamers of cTn-I is provided by the N-domains of PCN because of its structural similarity with the N-densities of the nucleotides. The delocalized pi-electrons in the C_3_N_3_ rings of the PCN *s*-triazine modules enable pi-stacking interactions with the cTn-I aptamers.

### 3.4. Determination of cTn-I

**Figure 8:**
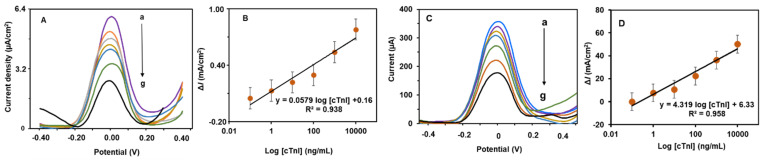
(**A**) SWVs of the PCN-RuNPs biosensor for various concentrations of cTn-I (a = 0, b = 0.1, c = 1.0, d = 10, e = 100, f = 1000, and g = 10,000 ng/mL) and (**B**) corresponding calibration plot. (**C**) SWVs of the PCN-NiMoO_4_ NRs biosensor for various concentrations of cTn-I (a = 0, b = 0.1, c = 1.0, d = 10, e = 100, f = 1000, and g = 10,000 ng/mL) and (**D**) corresponding calibration plot.

Next, quantitative determination of cTn-I was analyzed with the PCN-RuNPs aptasensor and the PCN-NiMoO_4_ NRs aptasensor in the presence of various concentrations of cTn-I. The SWV analysis of cTn-I levels was performed, from low nanomolar to high micromolar levels, and their corresponding curves are presented in Figure 8. Both the PCN-RuNPs aptasensor and the PCN-NiMoO_4_ NRs aptasensor showed a consistent decrease in the current signal when higher concentrations of cTn-I reacted with the aptamer. Calibration plots were derived by plotting normalized current response against the logarithmic concentration of cTn-I. The PCN-RuNPs aptasensor displayed a linear range of 0.1–10,000 ng/mL, a sensitivity of 0.0579 mA/(ng/mL)/cm^2^, and a detection limit (LOD) of 50 pg/mL. The PCN-NiMoO_4_ NRs aptasensor showed a linear range of 0.10–10,000 ng/mL, a sensitivity of 0.0608 mA/(ng/mL)/cm^2^, and a LOD of 70 pg/mL. The sensitivity of the PCN-NiMoO_4_ NRs was slightly better than the PCN-RuNPs, which is due to the slight difference in the background current and signal-to-noise ratio of these two material systems. Overall, both the PCN-RuNPs and PCN-NiMoO_4_ NRs aptasensors showed clinically useful analytical parameters. The analytical parameters are comparable with existing cTn-I biosensors (Table 1). In comparison to cTn-I sensors made using conventional electrodes, such as GCE [34] and ITO [35], the use of LSGEs offer several benefits including low-cost, flexibility, mass production, and disposability. However, the synthesis of PCN-RuNPs and PCN-NiMoO_4_ NRs depends on multiple lengthy synthesis steps, which is limiting their widespread application. Future research will focus on developing a fast, one-pot synthesis of nanomaterials.

**Figure 9:**
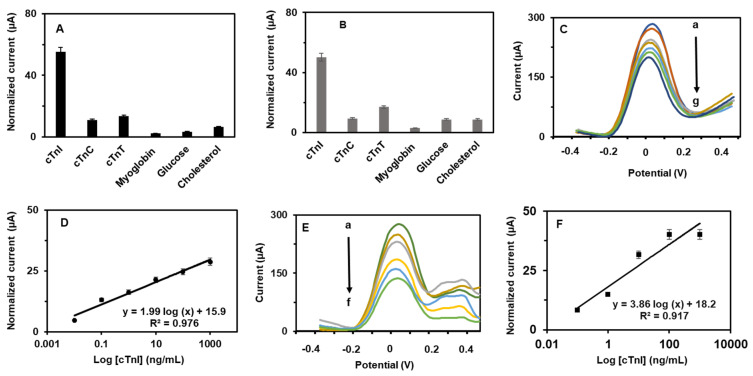
(**A**) Selectivity of PCN-RuNPs aptasensor and (**B**) PCN-NiMoO_4_ NRs aptasensor. SWV responses of 100 pg/mL cTn-I co-existed with 100 pg/mL cTnT, myoglobin, cholesterol, and glucose were recorded. (**C**,**D**) Practicality of PCN-RuNPs aptasensor and (**E**,**F**) PCN-NiMoO_4_ aptasensor in cTn-I-spiked human serum samples; a = 0, b = 0.1, c = 1.0, d = 10, e = 100, and f = 1000 ng/mL cTnI and their calibration plot.

Next, the selectivity of the PCN-RuNPs and PCN-NiMoO_4_ NRs aptasensors was examined in the presence of several potentially interfering biomolecules, including cTnT, cholesterol, myoglobin, and glucose (Figure 9). The normalized responses of the other tested molecules were less than 5%, indicating the appreciable selectivity of the aptasensor. The selectivity nature of the biosensors entirely originated from the specificity of the aptamer sequence that was exclusively made for cTn-I. Next, the reproducibility of the aptasensors was tested. Five PCN-RuNPs aptasensors were prepared individually and their biosensing performance towards cTn-I was tested. The R.S.D. value of these five readings was less than 4.7%, suggesting the acceptable reproducibility of the sensor. Similar results were observed for the reproducibility of five individual PCN-NiMoO_4_ NRs aptasensors. Next, the storage stability of the biosensors was tested by recording their cTn-I sensing performance for a week, one reading per day. The sensors were stored at 4 °C, maintaining a humid environment when not in use. About 91.3% and 89.5% of the initial signal responses were retained after six days of use for the PCN-RuNPs and PCN-NiMoO_4_ NRs, respectively, suggesting acceptable stability. The aptamers were effectively attached to the nanomaterial-modified electrode, and the immobilization was consistent between different electrodes, providing stable and reproducible signals on multiple electrodes. The practical utility of the PCN-RuNPs aptasensor and the PCN-NiMoO_4_ NRs was demonstrated in human serum samples (Figure 9C–F). Three different quantities of cTn-I were spiked into the serum and the spiked samples were analyzed by SWV. A significant decrease was noted when cTn-I-spiked samples were incubated with the aptasensors. The spiked levels of cTn-I were calculated from the normalized current. Thus, the PCN-RuNPs aptasensor and the PCN-NiMoO_4_ NRs aptasensor were found to be practically useful for real-world applications. It is worth mentioning that the use of a disposable LSGE as an electrode holds direct clinical applications [40].

## 4. Conclusions

Functionalized porous graphitic carbon nitride nanomaterial-based 2D nanocomposites are excellent materials to use in fabricating highly sensitive and selective cTn-I biosensors. The 2D layered structure, porous structure, defect sites, and dopants of the PCN materials are found to be highly relevant for accommodating aptamers and establishing facile aptamer-analyte interactions. PCN-RuNPs and PCN-NiMoO_4_ nanomaterials have appreciable surface properties, electrochemically active surface areas, and biosensing properties. Their distinctive microstructures and nanostructures offer locations for immobilizing significant numbers of active aptamers. PCN-RuNPs and PCN-NiMoO_4_ aptasensors can detect cTn-I in the wide range of 0.1–10,000 ng/mL. The LOD of cTn-I detection was 70.0 pg/mL for the PCN-RuNPs aptasensor and 50.0 pg/mL for the PCN-RuNPs aptasensor. The sample volume of 10 µL is sufficient for practical analysis, which is useful for analyzing clinical samples. Furthermore, the aptasensors are selective and practically feasible in human serum samples.

## Figures and Tables

**Table 1:** Aptasensing performance of PCN-RuNPs and PCN-NiMoO_4_ NRs aptasensors in previous reports.

Material	Type	Technique	Linear Range (ng/mL)	LOD (pg/mL)	Real Sample
Porous graphene oxide/GCE [34]	Immunosensor	EIS	0.1–10	70.0	Clinical samples
Zinc ferrite/LSGE [36]	Aptasensor	SWV	0.001–200	1.0	Serum
magnetic metal organic frameworks Fe_3_O_4_@UiO-66/Cu@Au/SPGE [37]	Dual-aptasensor	DPV	0.05–100	16.0	Serum
Manganese oxide-reduced graphene oxide/ITO [35]	Immunosensor	EIS	0.008–20	8.0	Serum
cTnI imprinted polypyrrole/boron nitride quantum dots/GCE [38]	MIP sensor	DPV	0.01–5.00	0.5	Blood serum
Au electrode/Ab_1_ (detection probe); HRP-Ab_2_-Au-COF (signal probe) [39]	Sandwich Immunosensor	DPV	0.005-10	1.7	Human serum
PCN-RuNPs/LSGE [this work]	Aptamer	SWV	0.1-10,000	50	Serum
PCN-NiMoO_4_ NRs/LSGE [This work]	Aptamer	SWV	0.1-10,000	70	Serum

EIS: Electrochemical impedance spectroscopy; SPGE: Screen-printed gold electrode; Apt: Aptamer; Ab_1_: primary antibody; Ab_2_: Secondary antibody; COF: covalent organic frameworks; MIP: molecularly imprinted polymer.

## Data Availability

Not applicable.
